# PKC-Dependent Phosphorylation of eNOS at T495 Regulates eNOS Coupling and Endothelial Barrier Function in Response to G^+^ -Toxins

**DOI:** 10.1371/journal.pone.0099823

**Published:** 2014-07-14

**Authors:** Feng Chen, Sanjiv Kumar, Yanfang Yu, Saurabh Aggarwal, Christine Gross, Yusi Wang, Trinad Chakraborty, Alexander D. Verin, John D. Catravas, Rudolf Lucas, Stephen M. Black, David J. R. Fulton

**Affiliations:** 1 Department of Forensic Medicine, Nanjing Medical University, Nanjing, Jiangsu, China; 2 Vascular Biology Center Medical College of Georgia at Georgia Regents University, Augusta, Georgia, United States of America; 3 Department of Pharmacology, Medical College of Georgia at Georgia Regents University, Augusta, Georgia, United States of America; 4 Institute for Medical Microbiology, Justus Liebig University, Giessen, Germany; 5 Old Dominion University, Norfolk, Virginia, United States of America; Xi'an Jiaotong University School of Medicine, China

## Abstract

Gram positive (G^+^) infections make up ∼50% of all acute lung injury cases which are characterized by extensive permeability edema secondary to disruption of endothelial cell (EC) barrier integrity. A primary cause of increased permeability are cholesterol-dependent cytolysins (CDCs) of G^+^-bacteria, such as pneumolysin (PLY) and listeriolysin-O (LLO) which create plasma membrane pores, promoting Ca^2+^-influx and activation of PKCα. In human lung microvascular endothelial cells (HLMVEC), pretreatment with the nitric oxide synthase (NOS) inhibitor, ETU reduced the ability of LLO to increase microvascular cell permeability suggesting an endothelial nitric oxide synthase (eNOS)-dependent mechanism. LLO stimulated superoxide production from HLMVEC and this was prevented by silencing PKCα or NOS inhibition suggesting a link between these pathways. Both LLO and PLY stimulated eNOS T495 phosphorylation in a PKC-dependent manner. Expression of a phosphomimetic T495D eNOS (human isoform) resulted in increased superoxide and diminished nitric oxide (NO) production. Transduction of HLMVEC with an active form of PKCα resulted in the robust phosphorylation of T495 and increased peroxynitrite production, indicative of eNOS uncoupling. To determine the mechanisms underlying eNOS uncoupling, HLMVEC were stimulated with LLO and the amount of hsp90 and caveolin-1 bound to eNOS determined. LLO stimulated the dissociation of hsp90, and in particular, caveolin-1 from eNOS. Both hsp90 and caveolin-1 have been shown to influence eNOS uncoupling and a peptide mimicking the scaffolding domain of caveolin-1 blocked the ability of PKCα to stimulate eNOS-derived superoxide. Collectively, these results suggest that the G^+^ pore-forming toxins promote increased EC permeability via activation of PKCα, phosphorylation of eNOS-T495, loss of hsp90 and caveolin-1 binding which collectively promote eNOS uncoupling and the production of barrier disruptive superoxide.

## Introduction

Gram positive (G^+^) infections make up ∼50% of acute respiratory distress syndrome (ARDS) cases and *Streptococcus pneumoniae* infections account for 45% of all community-acquired pneumonia (CAP) cases. In individuals over 50 years of age, there are over 500,000 yearly cases of non-bacteremic pneumococcal pneumonia and over 25,000 pneumococcal-related deaths that result in a health care burden exceeding $5 billion dollars[1]. Despite considerable investigation, there are currently no effective therapeutics for ARDS- and CAP- related pulmonary barrier dysfunction. These facts provide a strong rationale for more intensive research into the molecular mechanisms of endothelial barrier regulation.

CAP is accompanied by extensive permeability edema, characterized by a disruption in endothelial cell (EC) barrier integrity [2]. Despite the extensive use of potent antibiotics and aggressive intensive-care support, the mortality rate in CAP remains unacceptably high [3]. A major complication with these infections is the endothelial cytotoxicity and edema induced by bacterial virulence factors. Pneumolysin (PLY, S. pneumoniae) as well as its family member listeriolysin-O (LLO, Listeria monocytogenes) are members of the family of cholesterol-dependent pore-forming cytolysins (CDCs) [4] and are released from G^+^ bacteria upon cell lysis. Bactericidal antibiotics can promote significant release of G^+^-toxins and cause extensive and enduring injury even in a sterile lung. These thiol-sensitive gram positive (G^+^) virulence factors oligomerize in the presence of cholesterol to form plasma membrane pores that not only stimulate calcium entry in various cell types but also stimulate phospholipase C and protein kinase C alpha (PKCα) as we and others have recently shown [5]–[7]. Listeria infections are most commonly associated with food-borne diseases but can result in severe and often fatal pulmonary diseases that are also characterized by permeability edema [8]. Despite the greater appreciation of the importance of G^+^ toxins, the mechanisms by which PLY and LLO induce endothelial barrier disruption are poorly understood.

The liberation of nitric oxide (NO) from the vascular endothelium has been well established as an important regulator of the permeability of the microcirculation. In the lung, numerous studies have shown that endothelial nitric oxide synthase (eNOS) is barrier protective [9], [10]. However, others have shown a facilitative role for eNOS in the actions of edemagenic agents and in eNOS knockout mice the ability of VEGF to induce microvascular permeability is reduced [11]. These disparate findings suggest that eNOS can have multiple roles in the regulation of endothelial permeability and this is influenced by the amount of NO, vascular bed and agonist [12]. The role of eNOS in the loss of endothelial barrier function in response to G^+^ toxins is poorly defined and a goal of the current study.

The activity of eNOS is strongly influenced by a number of post-translational mechanisms including protein phosphorylation [13]. eNOS is phosphorylated on at least 7 sites and serine residues, S615, S633 and S1177 (human isoform) lie within the two major auto-inhibitory domains of the C-terminus of eNOS and work collectively to increase enzyme activity [14]. In contrast to the positive regulatory phosphorylation sites, eNOS is negatively regulated by the phosphorylation of Threonine 495 (T495) [15]–[17] and endothelial agonists that activate eNOS induce the dephosphorylation of this site [15]–[17]. Increased phosphorylation of T495 (P-T495) is stimulated by activators of protein kinase C such as PMA and is prevented by inhibitors of conventional protein kinase C isoforms [15], [17]. The mechanism by which P-T495 modifies eNOS activity is not fully understood and studies using phospho-mutants of eNOS have shown that the P-T495 can “uncouple” eNOS, reducing the synthesis of NO and increasing superoxide production [18]. The production of superoxide from eNOS reduces NO-dependent signaling in two ways. Firstly, increased superoxide production comes at the expense of NO synthesis and secondly, superoxide interacts avidly with NO in a diffusion limited reaction to form peroxynitrite [27]. Peroxynitrite is a potent oxidant and has biological effects that are distinct from that of NO (nitration versus S-nitrosylation). The role of eNOS phosphorylation or uncoupling in response to G^+^ toxins is not resolved.

Protein: protein interactions have also been well documented in their ability to regulate eNOS activity. Hsp90 is a molecular chaperone that binds to numerous client proteins and regulates protein folding and post-translational activity [19]. The binding of hsp90 to eNOS has been shown to facilitate NO production and regulate the fidelity of eNOS synthesis whereby a loss of hsp90 binding can promote the increased synthesis of superoxide [20], [21]. Caveolin-1 is another well characterized protein that dynamically interacts with eNOS [22]–[24]. In contrast to hsp90, caveolin-1 is a negative regulator of eNOS activity but it does share a recently discovered ability to influence eNOS fidelity by sequestering and inhibiting uncoupled enzyme [25]. The ability of G^+^ toxin-to regulate eNOS phosphorylation and protein: protein interactions has not been studied. Therefore, the goal of this study was to elucidate the mechanisms by which the G^+^ toxins alter endothelial barrier integrity with a focus on the regulation of eNOS phosphorylation, protein: protein interactions and enzyme fidelity.

## Materials and Methods

### Cell culture

HLMVEC were isolated and cultured in-house as described previously [26] or from commercial sources (Lonza) and grown in Endothelial Growth Medium-2-Microvessel (EGM-2MV) consisting of defined growth factors and supplemented with additional FBS up to 5% final concentration (Lonza). Cells were grown at 37 °C in 5% CO2 incubator and used from passage 2–8. COS-7 cells were cultured in Dulbecco's modified Eagle's medium (Invitrogen, Carlsbad, CA) containing L-glutamine, penicillin, streptomycin, and 10% (v/v) fetal bovine serum. Cells were transfected using Lipofectamine 2000 reagent (Invitrogen) as described previously[27].).

### Antibodies and reagents

Antibodies against phosphorylated PKC substrates, eNOS P-T495, eNOS P-S1177, PKCα were purchased from Cell Signaling. GAPDH (Santa Cruz) and eNOS, hsp90 and caveolin-1 were obtained from BD biosciences. S-ethylisothiourea (ETU) and Gö 6976 were obtained from Sigma-Aldrich.

### Bacterial toxins

LPS-free LLO and PLY were expressed and purified from the wild type *L. innocua* 6a strain, as described previously[5], [28].

### Endothelial permeability

Measurements of trans-endothelial resistance (TER) in HLMVEC, an index of changes in permeability [29], were performed as described previously [30]. Approximately 60,000 cells were seeded per well in a 8W10E array. Media was changed at 24h and again to serum free media at 48h, prior to the addition of G^+^ toxins. Resistance was measured using the ECIS Zθ model and normalized to each well's value at 0h.Toxins were applied when the resistance was stable between 1000-1300 ohms at a frequency of 4000Hz and the capacitance was between 22-29 nanofarads. Cells were preincubated with ETU or Gö 6976 for 0.5h prior to the addition of toxins.

### Co-immunoprecipitation and Western blotting analysis

Cells were lysed on ice in 20 mM Tris-HCl (pH 7.4), 1% Triton X-100, 100 mM NaCl, 1 mM Na3VO4, 10 mM NaF, and 1% protease inhibitor cocktail (Sigma). Soluble extracts were incubated for 2 h at 4°C with relevant antibodies: anti-eNOS (BD Biosciences) and a negative isotype control mouse immunoglobulin (IgG) (Santa Cruz Biotechnology), and complexes precipitated with protein A/G agarose (Santa Cruz Biotechnology). Western blotting was performed as described previously using anti-GAPDH, eNOS, P-T495 eNOS, P-1177 eNOS, phosphorylated PKC substrate, hsp90 and caveolin-1.

### Transient knockdown of PKC gene with siRNA

The siRNA targeting PKCα (siRNA ID: s11094) was obtained from Applied Biosystems. Validated control and targeting siRNA were transfected into HLMVEC using siPORTTM Amine (Applied Biosystems).

### Measurement of Nitric Oxide and Peroxynitrite

The accumulation of nitrite in the medium was measured by NO-specific chemiluminescence (Ionics). Background levels of nitrite in control transfected cells were subtracted. The formation of ONOO^-^ was determined by the ONOO^−^-dependent oxidation of dihydrorhodamine (DHR) 123 to rhodamine 123 as we have previously described [31]. Transfected cells were replated onto black tissue culture-treated 96-well plates (Thermo Fisher Scientific) at a density of 5 × 104 cells/well. Medium 200 (phenol red-free) was added containing 5 µmol/L DHR 123. After 60-min incubation at 37°C, the fluorescence of rhodamine 123 was measured by excitation 485 nm, emission 545 nm using a POLARstar reader. Results are expressed as % DHR oxidation compared to control transfected cells.

### Measurement of Superoxide

Superoxide generation in intact cells was measured using electron paramagnetic resonance (EPR) measurements as described previously using the spin probe 1-hydroxy-3-methoxycarbonyl-2,2,5,5-tetramethylpyrrolidine·HCl (CMH; Alexis Biochemicals)[32]. HLMVEC were incubated with 20 µl of spin-trap stock solution consisting of CMH [20 µM in Dulbecco's PBS (DPBS) plus 25 µM desferrioxamine (Calbiochem) and 5 µM diethyldithiocarbamate (Alexis Biochemicals)] prior to addition of G^+^ toxins for 45-min at 37°C. Cells were then detached and pelleted by centrifugation (500 × g). The cell pellet was washed and suspended in a final volume of 35 µl of DPBS, loaded into a 50-µl capillary tube, and analyzed with a MiniScope MS200 EPR (Magnettech, Berlin, Germany) at a microwave power of 40 mW (modulation amplitude of 3,000 mG, and modulation frequency of 100 kHz). The amplitude of EPR spectra were measured using ANALYSIS software (version 2.02; Magnettech).

### Statistical analysis

Data were reported as mean ± SE and statistical analyses performed using Instat software (GraphPad Software Inc., San Diego, CA) with a two-tailed student's t-test or ANOVA with a post-hoc test where appropriate. Differences were considered as significant at p < 0.05.

## Results

### The ability of G^+^-toxins to disrupt endothelial permeability is dependent on PKC and NO signaling

**Figure 1:**
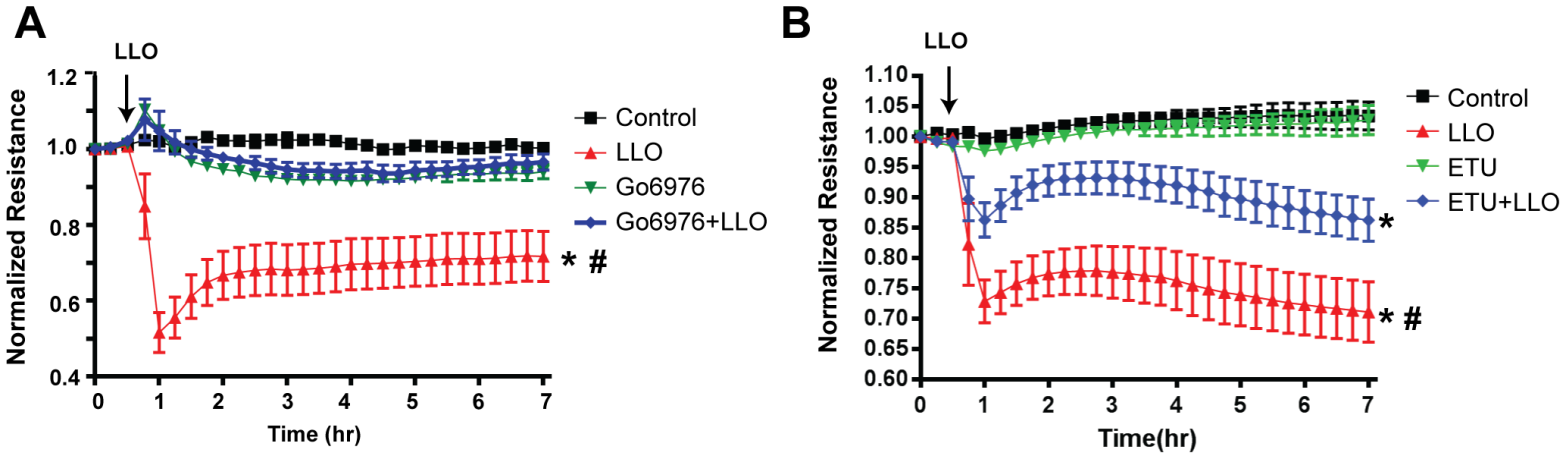
Inhibition of either eNOS or Protein Kinase C (PKC) ameliorates endothelial barrier disruption to G^+^-toxins. Confluent human lung microvascular endothelial cells (HLMVEC) were pretreated with and without (**A**) the PKCα inhibitor, Go6976 (1 µM,) or (**B**) the NOS inhibitor (ETU, 100 µM) and stimulated with the Gram positive bacterial (G^+^) toxin, listeriolysin (LLO, 250ng/ml). Changes in trans-endothelial resistance (TER) were recorded over time. (n = 6),* p<0.05 versus control, # p<0.05 versus ETU or Go6976.

HLMVEC were cultured to confluence on integrated gold-plated electrodes and transendothelial electrical resistance (TER) monitored until a stable plateau was attained. Cells were then pretreated with inhibitors of nitric oxide synthase (ETU [33], 100 µM, 30 mins) or protein kinase C (Gö 6976, 10 µM, 30 mins). Following the reacquisition of a stable baseline, the G^+^ positive toxin, LLO (250 ng/ml) was administered in serum free media and decreases in TER are reflective of increased permeability of the endothelial cell monolayer [29]. In cells pretreated with ETU or Gö 6976 there was a significant reduction in the ability of LLO to disrupt the endothelial barrier (**Figure1A-B**) suggesting functional roles for PKC and endothelial nitric oxide synthase (eNOS) in the ability of G^+^ toxins to disrupt the endothelial barrier.

### G^+^-toxins induce superoxide release from HLMVEC in a PKC and NOS-dependent manner

**Figure 2:**
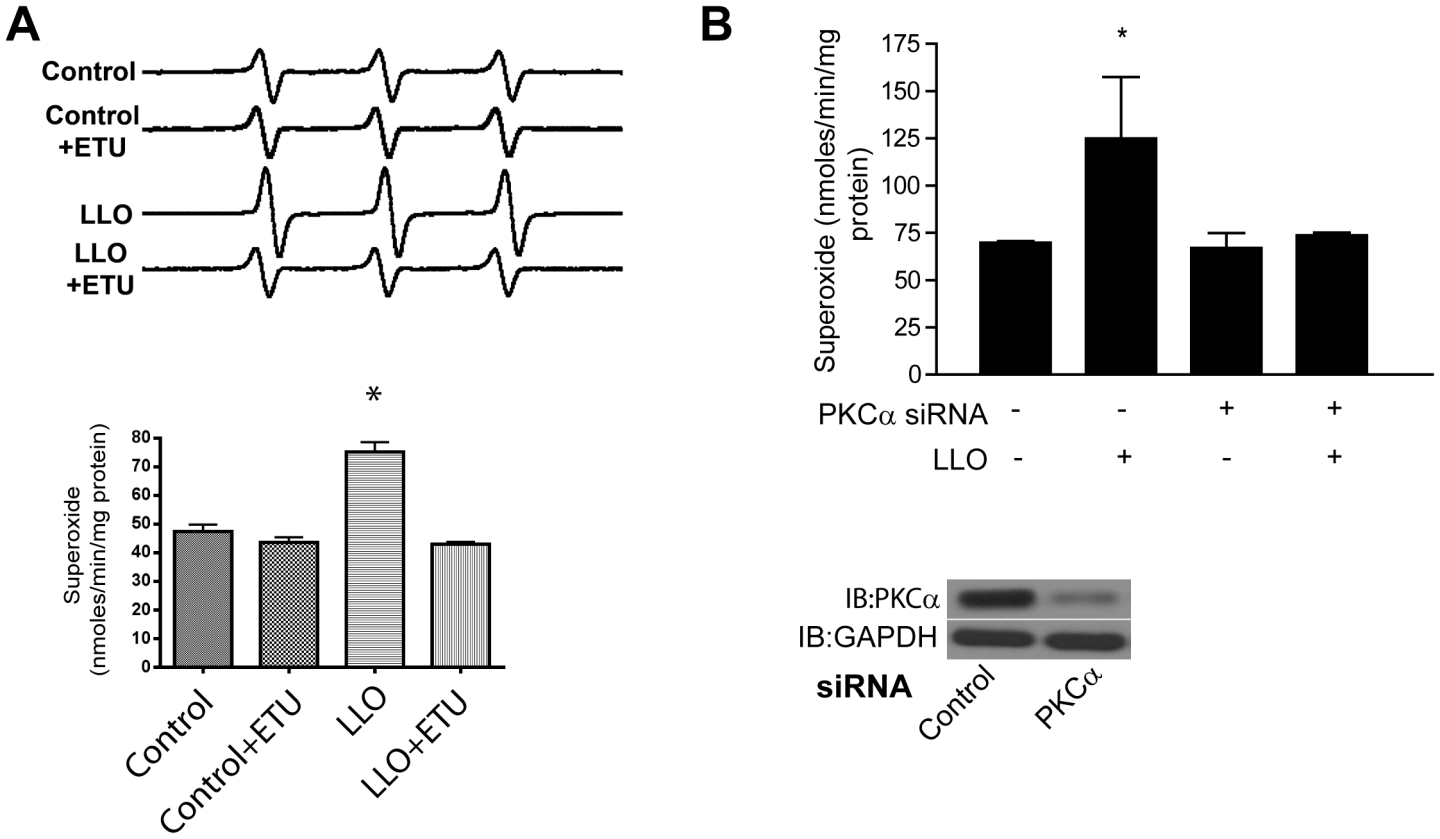
G^+^ -bacterial toxins stimulate endothelial superoxide production. HLMVEC were exposed to LLO (250 ng/ml, 30 min) in (**A**) the presence and absence of ETU (100 µM, representative trace shown in the upper panel, quantification in the lower panel) or (**B**) HLMVEC transfected with control or PKCα siRNA (60 nM). Superoxide was detected using the CMH (20 µM) spin probe and EPR. PKCα expression relative to GAPDH in HLMVEC lysates was determined by Western blot. n = 4–5. * p<0.05 versus control

Confluent HLMVECs were stimulated with LLO (250 ng/ml) and superoxide levels measured using EPR with the spin probe CMH. LLO induced the release of superoxide from HLMVEC and this ability was absent in cells pretreated with the NOS inhibitor (ETU, 100 µM) (**Figure 2A–B**). We next investigated the role of PKCα in HLMVEC transfected with either control siRNA (validated commercial control siRNA that does not target mammalian genes) or siRNA to PKCα. The ability of LLO (250 ng/ml) to promote superoxide release was significantly reduced in cells in which PKCα was silenced (**Figure 2C–D**) indicating that the ability of G^+^ toxins to induce superoxide release is dependent on both PKCα and eNOS.

### G^+^-toxins stimulate PKCα and eNOS T495 phosphorylation

**Figure 3:**
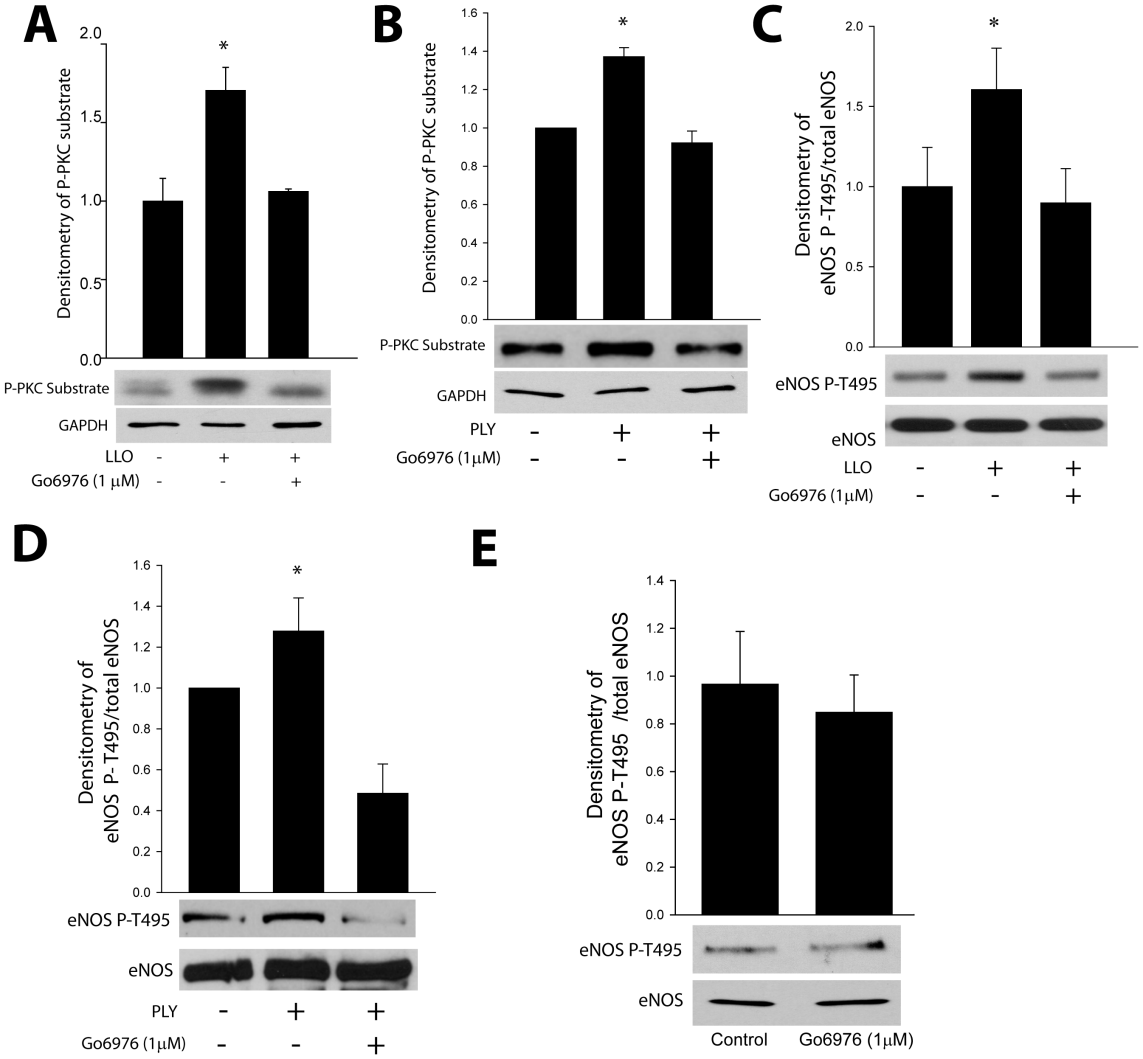
G^+^ -bacterial toxins stimulate the phosphorylation of PKC substrates and eNOS at T495. HLMVEC cells were pretreated with or without the PKCα inhibitor (Go6976, 1 µM) and stimulated with listeriolysin (LLO, 60 ng/ml) or pneumolysin (PLY, 30 ng/ml) for 30 min. The phosphorylation of (**A–B**) PKC substrates and (**C–D**) eNOS at T495 was determined using phosphorylation state specific antibodies. (n = 5). * p<0.05.

**Figure 4:**
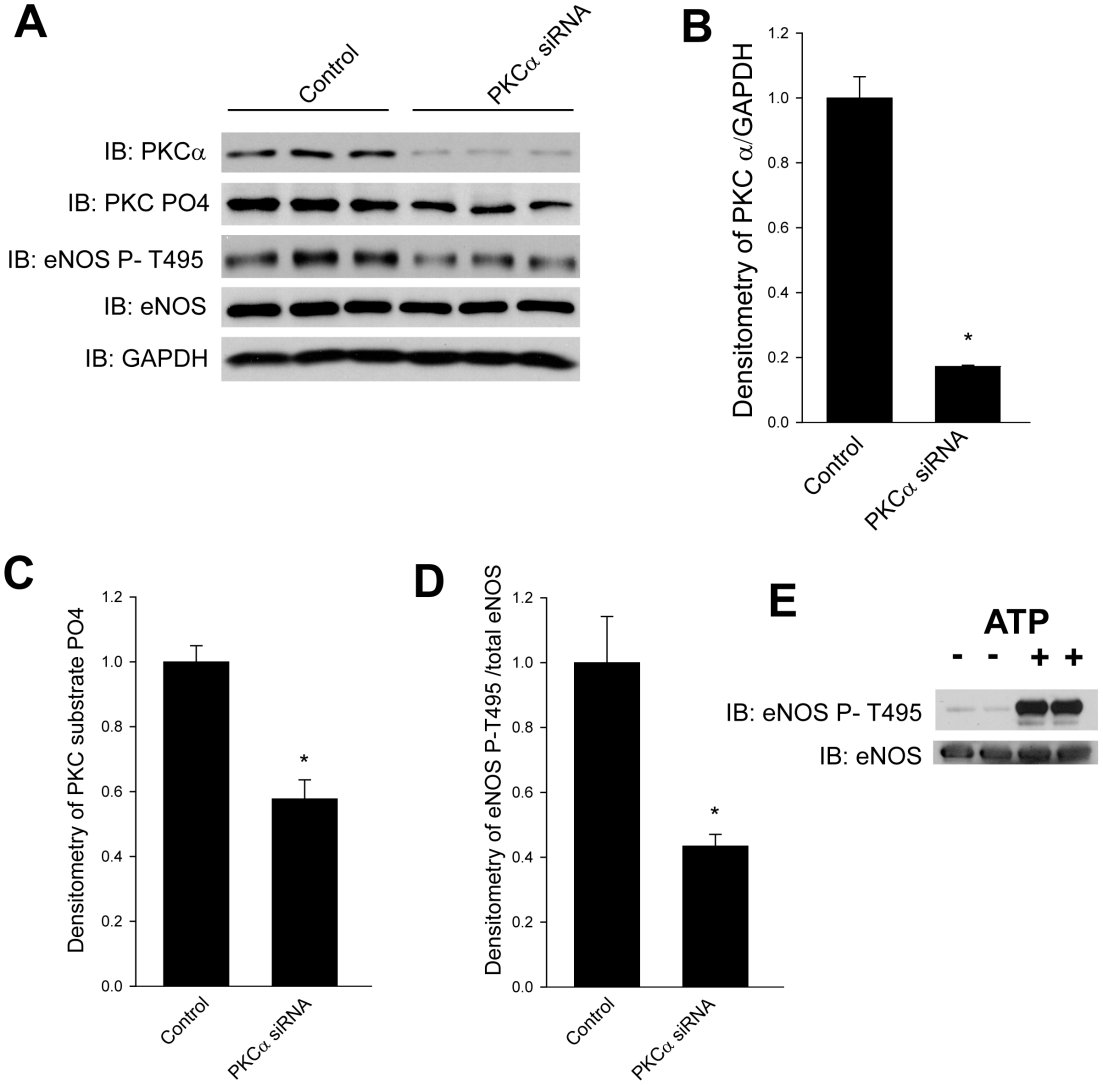
G^+^ -bacterial toxins utilize PKCα to stimulate eNOS T-495 phosphorylation. (**A**) HLMVEC were transfected with control or PKCα specific siRNA (60 nM), stimulated with PLY (30 ng/ml, 30 min.) the relative degree PKCα, phosphorylated PKC substrates, phosphorylated eNOS T495, total eNOS and GAPDH determined by Western blot. (**B**) Relative densitometry of PKCα relative to GAPDH, (**C**) phosphorylated PKC substrates (**D**) phosphorylated eNOS T495 relative to total eNOS. (**E**) In vitro kinase assay showing the ability of active recombinant PKCα to directly phosphorylate affinity purified human eNOS at T495. (n = 2–5). * p<0.05 versus control.

Confluent HLMVEC were pretreated with the PKC inhibitor Gö6976 and then exposed to LLO or PLY for 30 min. Cells were lysed and Westerns blots performed using antibodies that recognize the increased phosphorylation of PKC substrates as a measure of PKC activity. Both LLO and PLY increased the phosphorylation of PKC-substrates and this was significantly decreased by the PKC inhibitor Go6976 (**Figure 3A–B**). In parallel experiments, we next assessed the ability of LLO and PLY to increase the phosphorylation of eNOS at T495, a known PKCα phosphorylation site [17]. Using a selective eNOS T495 phosphorylation state specific antibody, we found that both LLO and PLY significantly increased eNOS phosphorylation at T495 and this effect was blunted in cells pre-treated with the PKC inhibitor (**Figure 3C–D**). There was no significant change in the basal phosphorylation of eNOS T-495 in the presence of Go6976 (**Figure 3E**). We next used a genetic approach to selectively silence PKCα in HLMVEC. HLMVEC were transfected with human specific PKCα siRNA or a validated control siRNA (as shown in **Figure 2D**). Cells were stimulated with PLY and immunoblotted for PKCα, phosphorylated PKC substrates, eNOS phosphorylated at T495, total eNOS and a loading control, GAPDH. The ability of PLY to promote increased PKC activity and eNOS T-495 phosphorylation was significantly blunted in cells where PKCα was silenced (**Figure 4A–D**). To verify that eNOS is a bonafide direct substrate for PKCα, we performed an *in vitro* kinase reaction using recombinant PKCα and affinity (ADP-sepharose) purified eNOS. We found that in the presence of ATP, eNOS was robustly phosphorylated by PKCα as determined by the relative levels of phosphorylated P-T495 eNOS to total eNOS (**Figure 4E**).

### Phosphorylation of T495 eNOS promotes uncoupling

**Figure 5:**
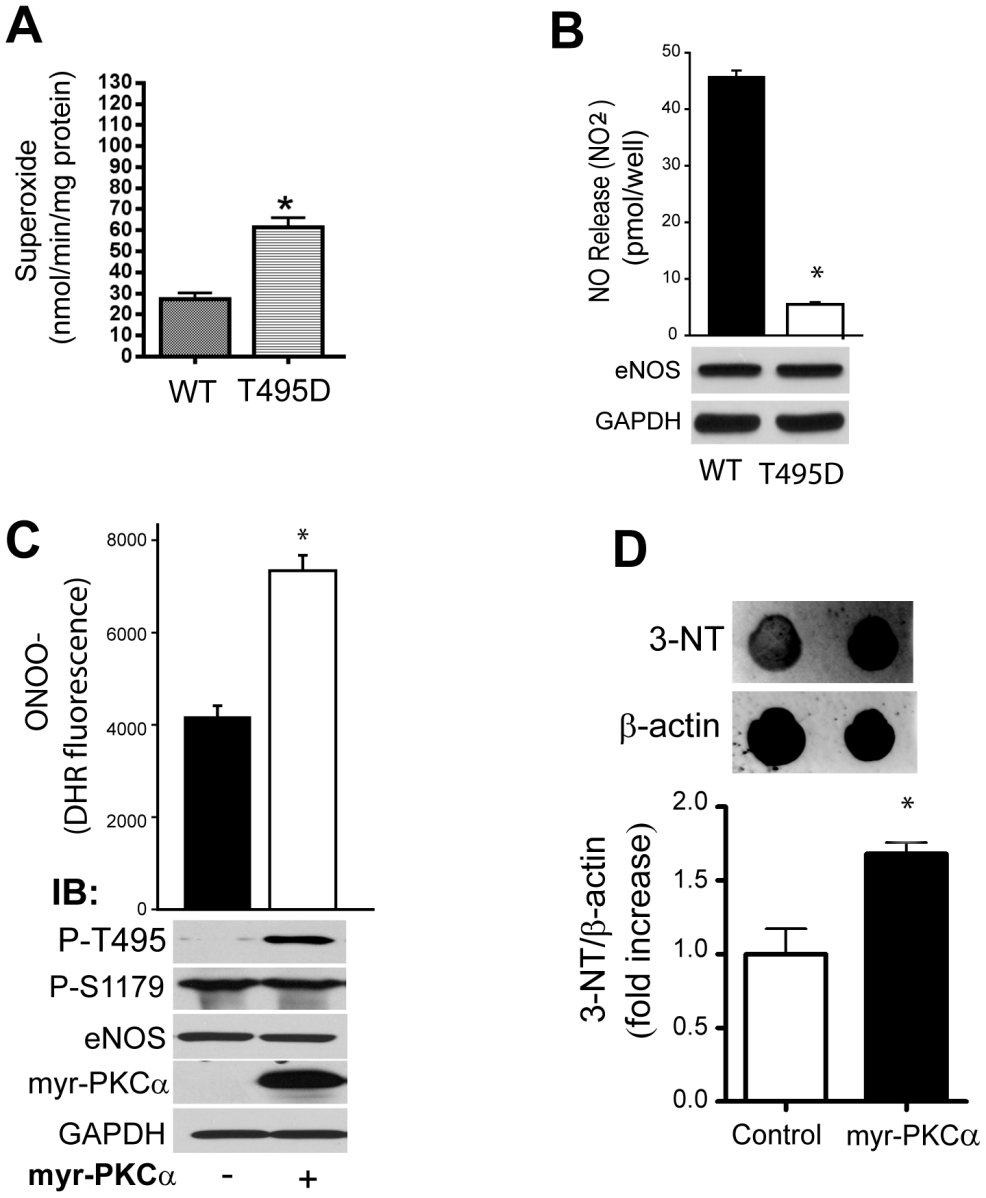
The PKCα-dependent phosphorylation of eNOS at T495 results in increased superoxide production. (**A**) Superoxide release as determined by EPR and (**B**) nitric oxide release measured by NO-specific chemiluminescence from COS-7 cells transfected with WT eNOS or the T495D phosphomimetic. The level of eNOS in transfected cells was determined by Western blot (lower panels). (**C**) Upper graph, relative production of peroxynitrite (ONOO-) as determined by DHR fluorescence in COS-7 cells co-transfected with WT eNOS with and without an active form of PKCα (myr-PKCα). Lower panels, the relative expression of phosphorylation of eNOS at T495 and S1179, total eNOS, expression of V5-tagged myr-PKCα and GAPDH as determined by Western blotting. n = 4–5. * p<0.05 versus control/WT.

To ascertain the functional consequences of phosphorylation of T495 on eNOS, we expressed aT495D phosphomimetic form of eNOS in COS-7 cells and measured the production of superoxide via EPR and the production of NO using NO-specific chemiluminescence relative to that from the wild type (WT) enzyme. The eNOS T495D phosphomimetic produced significantly higher amounts of superoxide and reduced amounts of NO compared to the WT enzyme at equal levels of expression (**Figure 5A, B**). To determine whether the increased phosphorylation of T495 on eNOS can result in enzyme uncoupling we co-transfected eNOS with an active form of PKCα. The co-expression of WT-eNOS and myr-PKCα resulted in robustly increased eNOS P-T495 whereas S1179 phosphorylation was unchanged (**Figure 5C**, lower panels). The phosphorylation of T495 was associated with increased peroxynitrite production as measured using DHR fluorescence (**Figure 5C**, upper graph) and a quantitative dot blot for 3-nitrotyrosine (**Figure 5D**).

### G^+^-toxins promote the dissociation of hsp90 and caveolin-1 from eNOS

**Figure 6:**
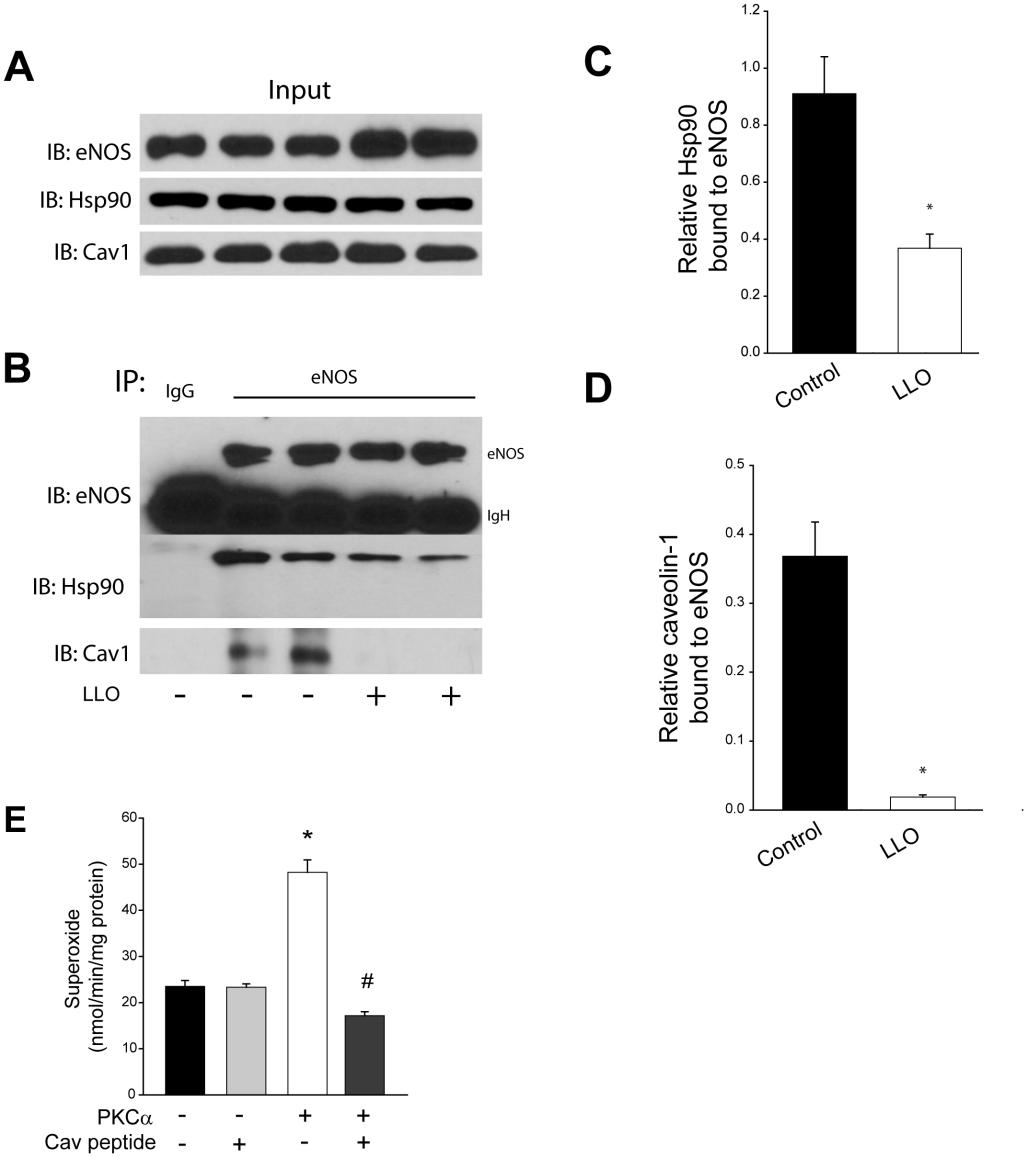
Gram^+^ toxin induced dissociation of caveolin-1 mediates the increase in eNOS-derived superoxide production. HLMVEC were treated with or without LLO (250 ng/ml, 30 min.) and (**A**) total levels of eNOS, hsp90 and caveolin-1(cav-1) determined by Western blot in cell lysates. (**B**) Lysates were subject to immunoprecipitation using either non-immune IgG or anti-eNOS and immune complexes immunoblotted for eNOS and associated hsp90 and caveolin-1. Relative densitometry of (**C**) hsp90 bound to eNOS or (**D**) caveolin-1 bound to eNOS. (**E**) In vitro superoxide production from eNOS affinity purified from COS-7 cells co-transfected with eNOS or eNOS with the active form of PKCα (myr-PKCα) in the presence and absence of the caveolin-1 scaffolding domain mimicking peptide (10 µM). n = 4–6. * p < 0.05

To determine the mechanism by which G^+^-toxins elicit eNOS uncoupling we monitored the relative binding of the eNOS associated proteins, hsp90 and caveolin-1. Both proteins have been shown to influence superoxide production from eNOS [21], [25] and it is not yet known whether G^+^-toxins influence their binding to eNOS. The addition of LLO to HLMVEC did not decrease the total cellular levels of eNOS, hsp90 or caveolin-1 (**Figure 6A**). To ascertain whether LLO impacts the relative binding of caveolin-1 and hsp90 to eNOS, eNOS was immunoprecipitated and the levels of bound hsp90 and caveolin-1 determined by Western blotting. We found that LLO induced a significant decrease in the association of eNOS with hsp90 and in particular caveolin-1 (**Figure 6B-D**). We next investigated whether the loss of caveolin-1-binding can impact the ability of PKCα to induce superoxide production from eNOS. COS-7 cells were transfected with eNOS and either a control plasmid (RFP) or eNOS and a constitutively active PKCα as shown in **Figure. 5C** and eNOS purified by affinity chromatography. *In vitro* superoxide production from purified eNOS was monitored by EPR in the presence of calmodulin, calcium and NADPH with and without the caveolin-1 scaffolding domain peptide. The production of superoxide from eNOS that was co-expressed with the active form of PKCα was greater than eNOS alone and the PKCα-stimulated increase in superoxide production was reversed by co-incubation with the caveolin-1 peptide (**Figure 6E**).

**Figure 7:**
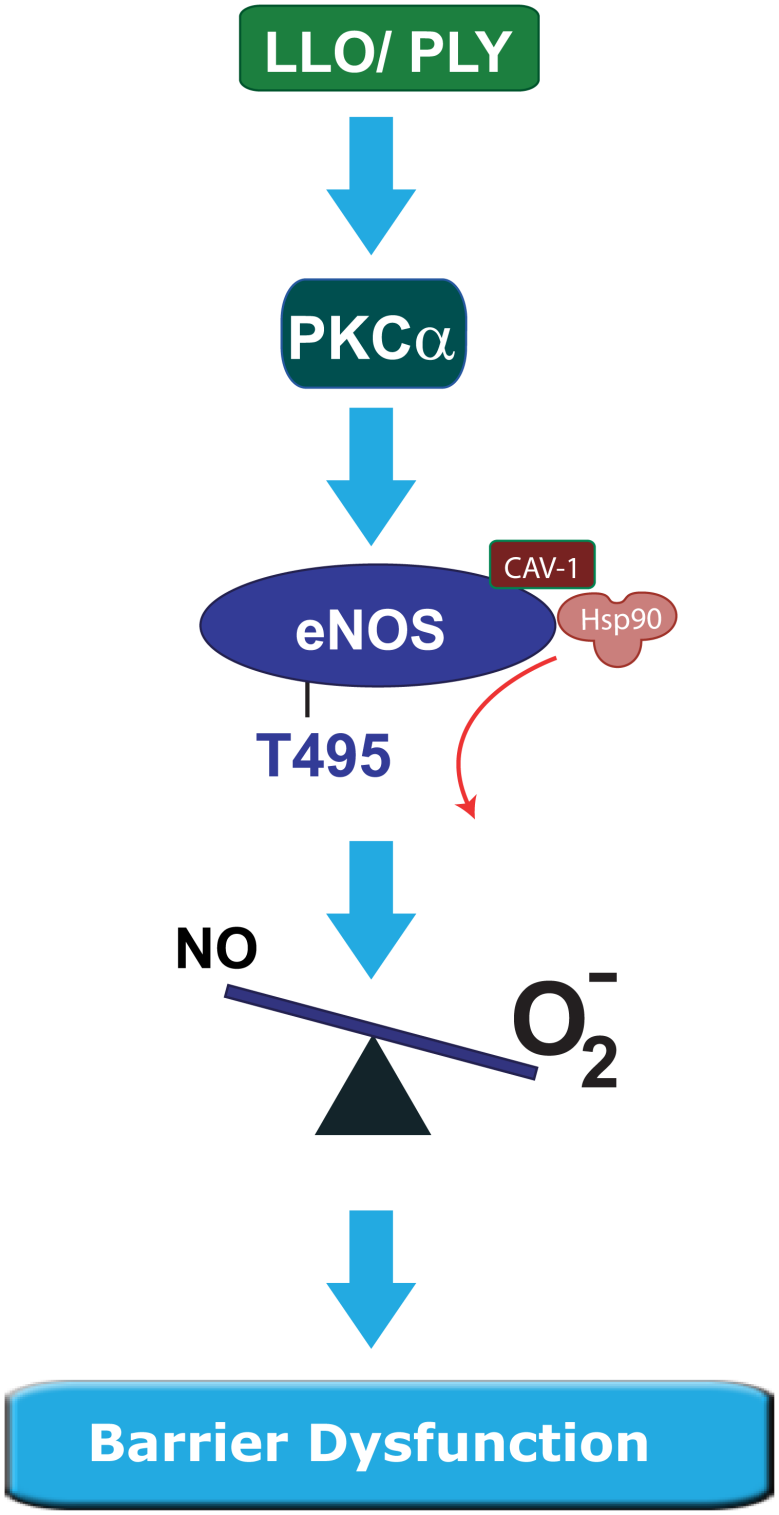
General overview of mechanisms proposed to regulate barrier function/dysfunction in response to the toxins PLY/LLO. The G^+^ exotoxins, LLO and PLY trigger the activation of PKCα. PKCα increases the phosphorylation of eNOS at T495 and decreases the association of caveolin-1 and hsp90 and alters the balance of NO/superoxide.

Collectively, these data suggest that LLO and PLY stimulate eNOS uncoupling in HLMVEC via the activation of PKCα. Active PKCα stimulates the phosphorylation of eNOS at T495 and together with reduced caveolin-1 and hsp90 binding this leads to a reduction in enzyme fidelity resulting in greater production of superoxide and peroxynitrite and a corresponding decrease in the levels of barrier protective NO (**Figure 7**).

## Discussion

Previous studies have shown that disruption of the pulmonary endothelial barrier to the G^+^-toxins, LLO and PLY involves the activation of PKC [5], [6]. The goal of the current study was to identify the downstream mechanisms by which PKC promotes disruption of the microvascular endothelial barrier. In HLMVEC we found that an inhibitor of nitric oxide synthases prevented the ability of G^+^-toxins to decrease transendothelial resistance, suggesting that eNOS was a crucial mediator of barrier dysfunction. Given that NO is generally considered to be barrier protective [10], [34]-[36], we investigated whether G^+^ toxins promote the synthesis of superoxide, which can also be produced from uncoupled eNOS. We found that the G^+^ -toxin, LLO promoted the PKCα and NOS-dependent production of superoxide. Subsequent studies revealed that the PKC and eNOS-dependent production of superoxide is connected by the ability of LLO/PLY to promote the PKC-dependent phosphorylation of eNOS at T495. To determine whether the phosphorylation of T495 can account for the increased superoxide production from eNOS we expressed the phosphomimetic T495D eNOS. Compared to the WT enzyme, the T495D eNOS produced more superoxide and less NO at equal levels of expression. Furthermore, an active form of PKCα stimulated the phosphorylation of T495 on eNOS and increased the production of peroxynitrite which is formed by the interaction of NO with superoxide. Lastly, LLO stimulated the dissociation of caveolin-1 and hsp90 from eNOS, events that have been shown to increase eNOS-derived superoxide [21], [25]. This mechanism was confirmed using an *in vitro* assay where affinity purified eNOS co-expressed with active PKCα produced more superoxide than the WT enzyme alone and the ability of a caveolin-1 scaffolding domain peptide to reverse the excess production of superoxide from eNOS co-expressed with PKCα. Overall our results support the ability of G^+^ toxins to disrupt the integrity of the HLMVEC barrier via the activation of PKCα, phosphorylation of T495 eNOS, loss of caveolin-1 and hsp90 binding and disruption of the NO/superoxide balance favoring the increased production of superoxide.

The ability of NOS inhibitors to protect against G^+^-toxin induced barrier disruption was a surprising observation. The consensus view is that eNOS-derived NO and exogenous NO protects against increased endothelial permeability in response to a number of agents including thrombin [34], hyperoxia [36], oxidants [37], PMA [38] and TxA2 [39] and also has an important role in maintaining barrier function under basal conditions [10]. In contrast, reactive oxygen species have been shown to promote disruption of the endothelial barrier [40]-[42]. However, it is less well appreciated that nitric oxide synthase inhibitors can protect against barrier disruption in response to stimuli that are known to promote superoxide production [43] and also that oxidants and reactive nitrogen species such as peroxynitrite can directly promote endothelial barrier dysfunction[44]. Recent evidence suggests that under the appropriate conditions and with certain stimuli, eNOS can produce increased amounts of superoxide and peroxynitrite at the expense of nitric oxide in a process termed eNOS uncoupling [45], [46].

It has previously been shown that G^+^-toxins induce superoxide production in neutrophils that express the NADPH oxidase, Nox2 [47] but whether G^+^-toxins stimulate superoxide from HLMVEC has not yet been studied. We found that LLO stimulated superoxide production in HLMVEC that was both PKCα and eNOS-dependent. The ability of PKCα to promote eNOS T495 phosphorylation suggested a possible mechanism for eNOS uncoupling. Previous studies have suggested that phosphorylation of eNOS at T495 can compromise enzyme fidelity [18]. Consistent with this, we found that a phosphomimetic T495D eNOS produces greater superoxide and reduced NO and that activation of PKCα can drive eNOS T495 phosphorylation and the increased formation of peroxynitrite. In contrast, Chen et al. showed that the direct *in vitro* phosphorylation of recombinant eNOS at T495 by PKCα does not result in increased superoxide production [48]. Reconciling these seemingly disparate findings are studies showing that changes in protein: protein interactions can influence eNOS fidelity and superoxide production. For example, Pritchard et al. showed that inhibition of the N-terminal ATP-dependent, protein folding activity of hsp90 and the subsequent loss of hsp90 binding to eNOS results in eNOS uncoupling and increased superoxide production [21]. More recently, Karrupiah et al. have shown that caveolin-1 binds preferentially to biopterin-deficient eNOS and reduces the amount of superoxide produced from uncoupled eNOS [25]. In the current study, we found that G^+^ toxins promote decreased hsp90, and in particular, caveolin-1 binding to eNOS. The ability of calcium-mobilizing agonists to promote the dissociation of eNOS and caveolin-1 has been shown by others [22], [23], [49]. It should also be noted that PLY, apart from directly affecting NO generation by eNOS, can also indirectly affect eNOS function, i. e. by means of increasing the activity of the eNOS competitor arginase 1[5].

Both LLO and PLY are pore forming toxins that stimulate calcium entry [5], [28], [47], [50], [51] and thus it is likely that elevation of intracellular calcium and enhanced calcium/calmodulin-binding to eNOS contributes to the ability of G^+^ toxins to promote the dissociation of caveolin-1. To determine whether loss of these proteins mediates the reduction in eNOS fidelity and increased superoxide production, we measured superoxide production from eNOS in an *in vitro* NOS activity assay in the presence and absence of a caveolin-1 peptide. The caveolin-1 peptide mimics the region of caveolin-1 that binds to eNOS [24], [52] and completely reversed the increased production of superoxide from eNOS activated by PKCα, which is consistent with the findings of other studies [25], [48]. Other studies have shown that microvascular permeability is increased in caveolin-1 knockout mice along with increased peroxynitrite and protein nitration and that these effects can be reversed with inhibition of eNOS and antioxidants[53]–[55]. Collectively, these results suggest that the loss of eNOS fidelity, mediated at least in part by decreased caveolin-1 binding, can promote increased endothelial permeability.

While our study supports a role for PKCα in the phosphorylation of eNOS on T495, it should be noted that other PKC isoforms and kinases can influence the phosphorylation of this site and this may be dependent on the cell type and stimulus. The phosphorylation of eNOS at T495 can be mediated by other PKC isoforms including PKCβ [56] and PKCδ [57] as well as other kinases such as AMPK [58] and Rho kinase [59]. It is not yet known whether LLO or PLY employ other PKC isoforms or kinases to induce endothelial barrier disruption. However, our data suggests that silencing PKCα alone is sufficient to suppress stimulated increases in superoxide and eNOS T495 phosphorylation. The mechanisms by which PLY and LLO activate PKCα are not fully elucidated. The ability of G^+^ pore forming toxins to mobilize calcium is well established [5], [28], [47], [50] and lanthanum chloride, an a specific inhibitor of calcium entry has been shown to reduce the barrier-disruptive effects of PLY in HLMVEC. G^+^ pore forming toxins, however, do not act strictly as calcium ionophores and have been shown to stimulate numerous signaling pathways including phospholipase PLA2 [60] and PLC [47]. PLY is known to bind to cell membranes in a cholesterol dependent manner that induces significant deformation of the membrane and a diverse population of transmembrane pores of different sizes and ion conductivity [61], [62]. Others have shown that PLY binding can promote the destabilization of membranes leading to aggregated vesicles and fusion of liposomes[63]. How this influences PLC and PKCα activity is not yet known but it is likely that the combination of altered membrane signaling in combination with robust calcium entry is sufficient to activate PKCα. The relative ability of the PKC inhibitor to protect against G^+^-toxin induced barrier disruption was much greater than the protection afforded by suppression of eNOS suggesting the possibility that additional mechanisms contribute to barrier disruption. In addition to eNOS, NADPH oxidases have been shown to be downstream targets of PKC [64]–[66]. It remains possible, and even likely, that NADPH oxidase isoforms in the vascular endothelium may be activated by PKCα in response to G+-toxins. This possibility will be explored in future studies.

Currently there are no established therapies for permeability edema associated with infection from G^+^- bacteria. Therefore a greater understanding of the mechanisms by which the G^+^-toxins, PLY and LLO evoke increases in microvascular permeability is a vital endeavor in the search for more effective therapeutics. Our study reveals novel roles for PKCα and eNOS uncoupling and strategies to prevent the loss of eNOS fidelity such as increasing caveolin-1 or the production of reactive oxygen species may be of significant benefit in reducing the morbidity and mortality of G^+^-induced pneumonia and pulmonary edema.
